# Balloon-Occluded Trans-Arterial Chemoembolization Technique with Alternate Infusion of Cisplatin and Gelatin Slurry for Small Hepatocellular Carcinoma Nodules Adjacent to the Glisson Sheath

**DOI:** 10.1155/2019/8350926

**Published:** 2019-05-09

**Authors:** Toshiyuki Irie, Nobuyuki Takahashi, Toshiro Kamoshida

**Affiliations:** ^1^Department of Radiology, Hitachi General Hospital, Japan; ^2^Department of Radiology, Tsukuba Memorial Hospital, Japan; ^3^Department of Hepato-Gastroenterology, Hitachi General Hospital, Japan

## Abstract

**Objective:**

It is difficult to control small hepatocellular carcinoma (HCC) nodules adjacent to the Glisson sheath (GS) by trans-arterial chemoembolization (TACE) probably due to multiple small tumor feeders directly branching from the trunk artery. The purpose of this study was to conduct a retrospective evaluation of a new TACE technique called the repeated alternate infusion of cisplatin solution and gelatin slurry distal to balloon occlusion (RAIB-TACE), for the treatment of small HCC nodules adjacent to GS.

**Materials and Methods:**

Small nodules less than 4 cm attached to proximal portion of the subsegmental to lobar level portal branch were retrospectively selected. Between January 2011 and April 2014, 29 nodules in 29 patients were treated by super-selective lipiodol TACE/balloon-occluded TACE (B-TACE) (Lip-TACE group). Since April 2014, treatment protocols for small nodules adjacent to GS were changed, and 14 nodules in 12 patients were treated by RAIB-TACE (RAIB-TACE group). In RAIB-TACE group, alternate infusion of cisplatin solution and sparse gelatin slurry (mixture of 80 mg of gelatin fragments and 20 mL of contrast medium) were repeated until arterial flow was ceased. In Lip-TACE group, lipiodol was used as drug carrier and dense gelatin slurry (mixture of 80 mg of gelatin fragments and 2 mL of contrast medium) as embolization material. Dynamic CT/MRI was obtained 1-3 months after TACE, and response of each nodule was evaluated basing on modified RECIST criteria.

**Results:**

In RAIB-TACE group, all 14 nodules (100%) were diagnosed as CR or PR. In Lip-TACE group, 18 of 29 (62.1%) were diagnosed as CR or PR. There was a statistically significant difference in objective response ratio between the groups (p=0.008, Fisher's test). Biloma (n=1) and benign stricture of the right hepatic duct (n=1) were seen in RAIB-TACE group. The biloma shrunk without treatment and the patient had no symptom, but the patient with biliary stricture repeated cholangitis and was treated by administration of antibiotics.

**Conclusion:**

The study results show that RAIB-TACE is more effective than lipiodol TACE/B-TACE for small hepatocellular carcinoma adjacent to GS. We speculate that one of the reasons to explain why Lip-TACE is inferior to RAIB-TACE is that viscous lipiodol or dense gelatin slurry could not flow into small tumor feeders effectively.

## 1. Introduction

Radiofrequency ablation (RFA) is widely performed to treat small hepatocellular carcinoma (HCC) nodules that are not in close contact with the Glisson sheath (GS). Small nodules <3cm could be treated by RFA if the nodules are 3 or less. The chance to perform super-selective TACE for small nodules that are not in close contract with GS is markedly decreased with the advancement of imaging technologies for needle puncture during RFA procedures [1–3]. Contrast medium for ultrasonography clearly depicted the target nodule during US-guided needle puncture, and transarterial infusion of lipiodol before RFA assisted CT-guided needle puncture. However, one limitation of RFA is that treatment of small HCC nodule adjacent to GS causes thermal damage on biliary tract [4, 5]. Thus, trans-arterial chemoembolization (TACE) is often performed to treat small HCC nodules adjacent to GS. But it is sometimes difficult to control HCC nodules adjacent to GS by super-selective TACE using a microcatheter [6] probably due to multiple small tumor feeders directly branching from the trunk artery. Development of a new TACE technique to treat small nodules adjacent to GS is an important issue.

Recently, effectiveness and safety of TACE technique without lipiodol using cisplatin solution and 1mm porous gelatin particles was reported for treatment of multiple nodules [7]. Our new TACE technique was developed by modifying and evolving this technique with use of a microballoon catheter and crushing the 1 mm particles [8] into smaller fragments (130-200 micron in length) to enhance anti-tumor effects. Infusion of cisplatin solution under balloon occlusion would increase its concentration in the nodules and smaller gelatin fragments would reach in the nodules [9, 10] while preventing collateral blood supply to the nodules. At first, we have performed this new balloon-occluded TACE (B-TACE) technique: repeated alternate infusion of cisplatin solution and sparse gelatin slurry under balloon occlusion (repeated alternate infusion B-TACE, RAIB-TACE) for lipiodol-TACE refractory nodules adjacent to GS to examine the effectiveness of this technique. Then, we performed RAIB-TACE to treat all small nodules adjacent to GS when TACE was indicated. The purpose of this study was to examine the efficacy of RAIB-TACE by retrospective analysis of treatment effect for small HCC nodules adjacent to GS.

## 2. Materials and Methods

### 2.1. Selection of Patients and Small HCC Nodules Adjacent to GS

HCC nodules attached with proximal portion of subsegmental to lobar level portal branch on dynamic CT/MRI were defined as those adjacent to GS. Recurred nodules after RFA/TACE were excluded from analysis. To simplify analysis, nodules 4 cm or more were also excluded because patients with nodules of >4cm in size were considered as poor responders to TACE [11, 12]. We considered the inclusion of large nodules in the analysis would increase a factor of TACE refractoriness other than tumor location (adjacent to GS or not), and the study design would be complicated.

Between January 2011 and April 2014, super-selective lipiodol TACE/B-TACE (Lip-TACE) was performed for nodules adjacent to GS regardless of the size. When biliary tract distal from the nodule was dilated, even slightly dilated, Lip-TACE was not performed for fear of biloma. Twenty-nine nodules in 29 patients were selected (Lip-TACE group) (Table 1).

Since April 2014, we changed the treatment protocols for small nodules adjacent to GS, and RAIB-TACE was performed for all when TACE was indicated (RAIB-TACE group). When the diameter of dilated biliary tract was less than that of the accompanied portal branch (slight dilatation), RAIB-TACE was performed. But when the diameter of biliary tract distal from the nodule was dilated more than that of the accompanied portal branch, RAIB-TACE was not performed because we speculated that the risk of biloma would be higher. Fourteen nodules in 12 patients were selected. Of these, 5 nodules in 5 patients (5/14, 35.7 %) were associated with slight biliary dilatation distal from the nodule (Table 1). 

### 2.2. Preparation of Embolization Materials for Lip-TACE and RAIB-TACE

Spherical porous gelatin particles 1 mm in diameter (Gelpart®, Nipponkayaku, Tokyo) were commercially available while preserved in a glass bottle containing 80 mg of particles. Immediately before use, particles were mixed with nondiluted contrast medium (Oypalomin, Iopamidol 300, Fuji Pharma., Tokyo) to crush by pumping method between 2 small size syringes (2.5 mL in capacity) through a partially opened 3-way stopcock. The diameter of tumor feeding artery at the entry site into the nodule was often less than 1 mm [13], and we considered that use of original size particles would cause proximal embolization. Thus, gelatin slurry containing smaller fragments (136.8 ± 60.8 to 261.4 ± 110.3 *μ*m, in wet state, 16 trials of crushing) were created to enhance antitumor effect by crushing the original size particles [8]. Dense gelatin slurry (mixture of 80 mg of gelatin fragments and 2mL of contrast medium) was used for Lip-TACE, and sparse gelatin slurry (mixture of 80 mg of gelatin fragments and 20mL of contrast medium) for RAIB-TACE.

### 2.3. Super-Selective Lipiodol TACE/B-TACE (Lip-TACE) Technique

**Figure 1:**
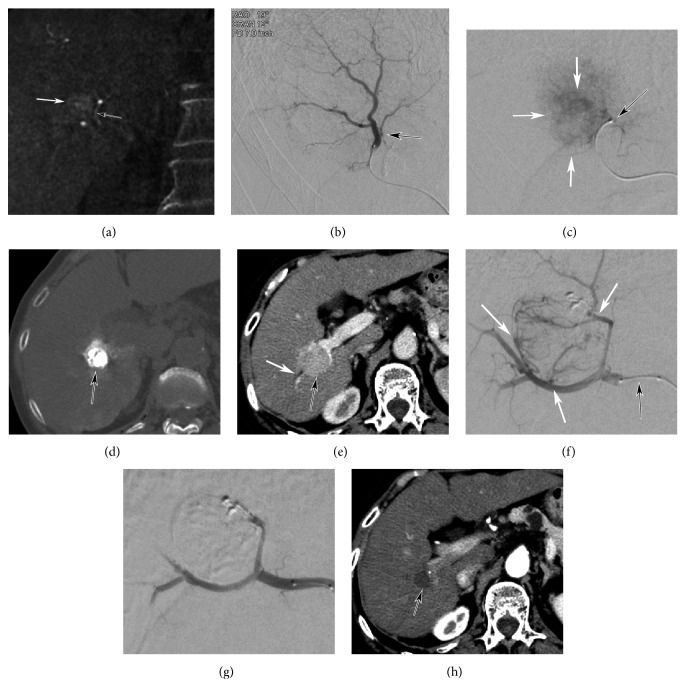
RAIB-TACE for HCC nodules adjacent to GS unresponsive to Lip-TACE in a 75-year-old patient. (a) Cone beam CT (coronal plane) with the injection of contrast medium in the replaced right hepatic artery from the superior mesenteric artery showing a small HCC nodule (white arrow) and a small tumor feeding artery (black arrow). (b) Appropriate projection DSA of the superior posterior subsegmental artery showing a small feeding artery (black arrow). (c) After advancing the microcatheter into the feeding artery (black arrow), DSA with the hand injection of contrast medium shows tumor stain (white arrows). (d) CT after Lip-TACE shows lipiodol accumulation within the tumor (black arrow). (e) Contrast-enhanced CT 12 weeks after Lip-TACE showing recurrent, enlarged HCC nodule (black arrow) associated with slight dilatation of a peripheral bile duct (white arrow) caused by tumor compression. (f) Selective DSA of the proximal posterior segmental artery (black arrow) showing recurrent tumor with multiple feeding arteries (white arrows). (g) DSA after RAIB-TACE showing occlusion of the tumor feeding arteries. (h) Dynamic CT two months after RAIB-TACE showing tumor shrinkage with no mass enhancement (black arrow).

**Figure 2:**
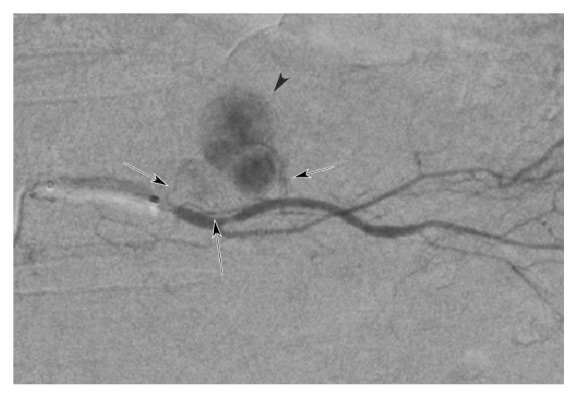
Multiple tortuous small tumor feeding arteries directly branching from the trunk artery. Appropriate projection DSA depicting tumor stain (black arrowhead) adjacent to GS and multiple tortuous small tumor feeding arteries directly branching from the trunk artery (black arrows).

Referring to DSA and transarterial cone beam CT/64-MDCT, small tumor feeders connected/adjacent to the nodules were searched on thin slice images. Appropriate projection of C-arm was detected to avoid and minimize overlapping of the trunk artery on tumor stain by using a workstation. Then, appropriate projection DSA was obtained via the trunk artery, and small tumor feeding arteries directly branching from the trunk artery were detected. A microcatheter (Pixie, Tokai Medical, Kanagawa /Sigma, Terumo, Tokyo/ Veloute, Asahi Intec, Nagoya) or a microballoon catheter (Logos, Piolax, Yokohama /Attendant delta, Terumo-Clinical, Kagamihara) was placed in tumor feeding artery and Lip-TACE was performed (Figure 1). When super-selective catheter placement in tumor feeding artery could not be done due to tortuosity/smallness of artery, a microballoon catheter was placed as close to the tumor stain or tumor feeding artery as possible and B-TACE was performed (Figure 2).

The emulsified anticancer drugs with 10 mL of lipiodol (Lipiodol 480 injection, Guerbet, Tokyo) were 10mg of doxorubicin (Adriacin injection, Kyowa Hakko, Tokyo) and 2-4 mg of mitomycin C (Mitomycin injection, Kyowa Hakko, Tokyo). Each drug was solved with 1 mL of contrast medium (Oypalomin 300, Fuji Pharm., Tokyo) before emulsifying (n=7). Since November 2011, solution of 10 mg of doxorubicin and 2 mg of mitomycin C (n=20) or that of 50 mg of cisplatin (n=2) was infused, followed by lipiodol suspension of miriplatin (Miripla, Dainippon Sumitomo Pharma, Osaka). Finally, dense gelatin slurry was infused until the tumor feeding arteries beyond the catheter tip were filled with the dense gelatin slurry.

### 2.4. RAIB-TACE Technique

Referring to DSA and transarterial cone beam CT/64-MDCT, proximal portion of subsegmental to segmental level artery adjacent to the nodule was detected for catheter placement. We placed a microballoon catheter at relatively proximal portion compared with lipiodol B-TACE. Immediately before injection, the sparse gelatin slurry in a medium size syringe (25 mL in capacity) was stirred by pumping method to prevent aggregation of fragments and was transferred into a microsyringe (1 mL).

Fine powder cisplatin (IA coal, cisplatin, Nipponkayaku, Tokyo) was solved with warmed saline as 1mg/mL and transferred into a large size syringe (50 mL in capacity) which was connected with a microsyringe and a microballoon catheter using a 3-way stopcock. To infuse cisplatin solution, transfer of cisplatin solution into a microsyringe from a large size syringe via the 3-way stopcock and rapid injection using a microsyringe were repeated. Under balloon occlusion of proximal portion of subsegmental to segmental level artery, 1-5 mL of cisplatin solution was infused followed by 0.5-1 mL of sparse gelatin slurry. Approximately 25 mg of cisplatin was used to treat one segment of the liver. Even under balloon occlusion, blood flow beyond the balloon catheter was maintained via collaterals [14–17]. This phenomenon was first reported by Sugai et al. [18]. Stasis of sparse gelatin slurry like branching tree beyond the balloon catheter and cease of blood flow were obtained after 1-5 repetitions of alternate infusion, and, then, the balloon was carefully deflated. When blood flow beyond the balloon catheter recurred after balloon deflation, alternate infusion was performed under balloon deflation until stasis of gelatin slurry was obtained.

We used sparse gelatin slurry for RAIB-TACE to prevent both aggregation of gelatin fragments and proximal embolization referring a paper disclosing how to inject drug-eluting beads to prevent both particle aggregation and proximal embolization [19].

### 2.5. Analysis of Follow up CT/MRI

Dynamic CT/MRI was performed 1-3 months after TACE for evaluation of response of each nodule basing on modified RECIST criteria [20].

### 2.6. Comparison of Tumor Response between the Groups, Analysis of Side Effects, and Angiographic Findings of Appropriate Projection DSA

The ratio of objective response (CR or PR) was compared between the groups using Fisher's test. Adverse events on blood chemistry data after TACE were assessed according to the National Cancer Institute Common Terminology Criteria (version 4.0). The analyzed factors were alanine aminotransferase (ALT) increase level and total bilirubin increase level. Major side effects such as biliary damages were investigated.

Depiction of tumor feeding arteries on appropriate projection DSA in Lip-TACE group was analyzed. However, appropriate projection DSA was not performed in RAIB-TACE group because catheter placement was not intended in small tumor feeding arteries directly branching from the trunk artery.

### 2.7. Efficacy of RAIB-TACE for Lip-TACE Refractory Nodules

For 9 nodules which showed SD or PD after Lip-TACE in Lip-TACE group, RAIB-TACE was performed. Of these, 2 were with slight biliary dilatation due to enlargement of the nodule after Lip-TACE (Figure 1). Tumor response was evaluated for these 9 nodules.

## 3. Results

In 29 nodules in Lip-TACE group, 17 nodules were diagnosed as CR, 1 as PR, 4 as SD, and 7 as PD, respectively. In 14 in RAIB-TACE group, 13 as CR, 1 as PR, respectively. Objective response ratio (CR or PR) of nodules in RAIB-TACE group (14/14, 100%) was higher than that in Lip-TACE group (18/29, 62.1%) with a statistically significant difference (Table 2, p=0.008, Fisher's test). There was no statistically significant difference in any factors of patient's profile between the groups (Table 1).

Bilirubin increase level was grade 1 or 2 in all patients of both groups. In one case of Lip-TACE group, grade 3 increase level of ALT was seen.But the rest all of both groups were level 1 or 2 increase level of ALT (Table 1). There were no statistically significant differences between the groups.

**Figure 3:**
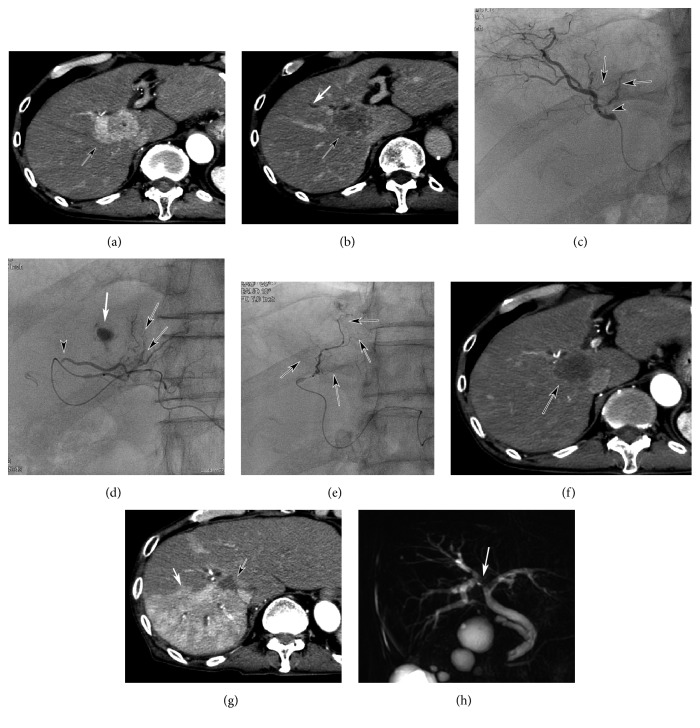
Right hepatic duct stricture complicating RAIB-TACE in a 79-year-old-patient with a HCC nodule adjacent to GS. (a) Arterial phase CT showing a hypervascular nodule (black arrow) adjacent GS. (b) Delayed phase of CT showing hypoenhancement of the tumor (black arrow) and slight biliary dilatation (white arrow) distal from the nodule. (c), (d), (e). Spot radiographies immediately after RAIB-TACE via the anterior segmental (c), caudate subsegmental (d), and medial subsegmental arteries (e). Stasis of sparse gelatin fragments with contrast medium is seen in multiple tumor feeders (black arrows, (c), (d), (f)). An anastomotic artery is seen between the caudate subsegmental (black arrowhead, (d)) and anterior segmental arteries (black arrow head, (c)). A vascular lake is also seen (white arrow, (d)). (f) CT obtained 2 months after RAIB-TACE showing CR of the tumor (black arrow). (g) CT obtained 13 months after RAIB-TACE showing shrinkage with no tumor enhancement (black arrow). The increased arterial perfusion (white arrow) to the posterior segment is due to due to cholangitis caused by stricture of the right hepatic duct. (h) MRCP showing benign stricture of the hilar bile duct (white arrow).

**Figure 4:**
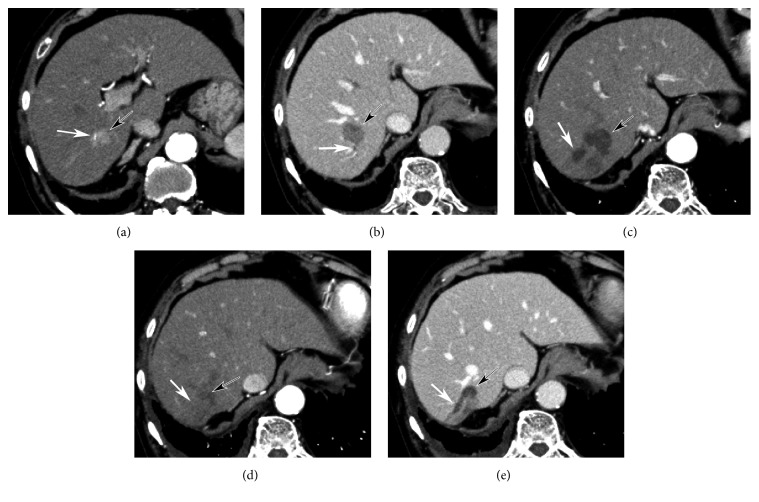
Biloma after RAIB-TACE in an 86-year-old patient with a HCC nodule adjacent to GS. (a) Arterial phase CT showing a hypervascular nodule (black arrow) attached to the superior posterior subsegmental artery (white arrow). (b) Portal phase CT at the different level showing slight biliary dilatation (white arrow) distal from the nodule (black arrow). (c) CT obtained 3 months after RAIB-TACE showing CR of the nodule (black arrow) and biloma (white arrow). No treatment was required for the biloma, as the patient remained asymptomatic. (d) and (e) CT obtained 10 months after RAIB-TACE revealing shrinkage of the biloma (white arrow) and no recurrence of the nodule (black arrow).

Biliary damage was seen in 2 patients of RAIB-TACE group, but not in any of Lip-TACE group. Both nodules were associated with slight biliary dilatation distal from the nodules (Figures 3 and 4). In one case, stricture of the right hepatic duct occurred with shrinkage of the nodule 8 months after RAIB-TACE (Figure 3). Because no tumor stain was seen on dynamic CT when this stricture occurred, subsequent tumor invasion could not be the cause. The cause of this biliary stricture was unknown, but we speculated that shrinkage of both the nodule and adjacent peri-biliary tissue would provoke this stricture because the stricture was limited to peri-tumor region and biliary dilatation occurred in nonembolized segment (Figure 3). This patient repeated cholangitis, and administration of antibiotics was required each time. In another case, biloma was seen (Figure 4). However, the patient was free from abdominal pain or fever, no administration of antibiotics was required, and the biloma shrunk without treatment.

In all 29 nodules in Lip-TACE group, appropriate projection DSA depicted one or more small tumor feeding arteries directly branching from the trunk artery. In 12 of 29 nodules, super-selective catheter placement in these could be achieved. In the rest of the 17 nodules, B-TACE was performed via the trunk artery, but lipiodol droplets were far larger than the diameter of small tumor feeding arteries and lipiodol droplets tended to flow into peripheral liver parenchyma on fluoroscopic observation.

**Figure 5:**
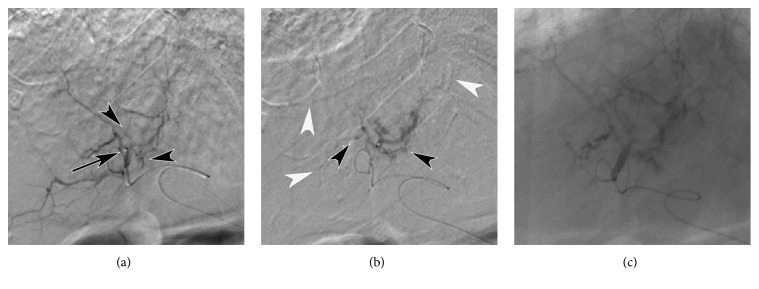
Arterial occlusion process of RAIB-TACE (same case of Figure 4). (a) DSA before RAIB-TACE showing a microballoon catheter at the superior posterior subsegmental artery (black arrow) and multiple small tumor feeders branching from proximal portion (black arrowheads). (b) DSA obtained after 2 repetitions of alternate infusion showing earlier embolization of distal branches (white arrowheads) and patent proximal tumor feeders (black arrowheads). (c) Spot radiography immediately after RAIB-TACE (5 repetitions of alternate infusion) showing stasis of contrast medium in both the distal branches and proximal tumor feeders.

Arterial occlusion process during RAIB-TACE procedure was observed under fluoroscopy and DSA (Figure 5). Distal branches were embolized at first, and, then, stasis of contrast medium containing sparse gelatin slurry was seen in small tumor feeders branching from proximal portion.

RAIB-TACE achieved CR (Figure 1) in 7 lip-TACE refractory nodules (78.8 %) and SD in 2 (Table 3). No major complication was seen in these.

## 4. Discussion

Objective response was achieved in all 14 nodules adjacent to GS (100 %) by RAIB-TACE. We had performed Lip-TACE to treat small nodules adjacent to GS before development of RAIB-TACE technique, and objective response was achieved in 18 of 29 nodules (62.1 %). The objective response ratio of RAIB-TACE was higher than that of Lip-TACE with a statistically significant difference. Thus, RAIB-TACE was superior to Lip-TACE for control of small nodules adjacent to GS. Moreover, RAIB-TACE achieved CR in 7 of 9 Lip-TACE refractory nodules. Basing on successful result of RAIB-TACE for treatment of Lip-TACE refractory nodules, we changed the protocols for treatment of small nodules adjacent to GS; we ceased Lip-TACE and performed RAIB-TACE for these since April 2014.

**Figure 6:**
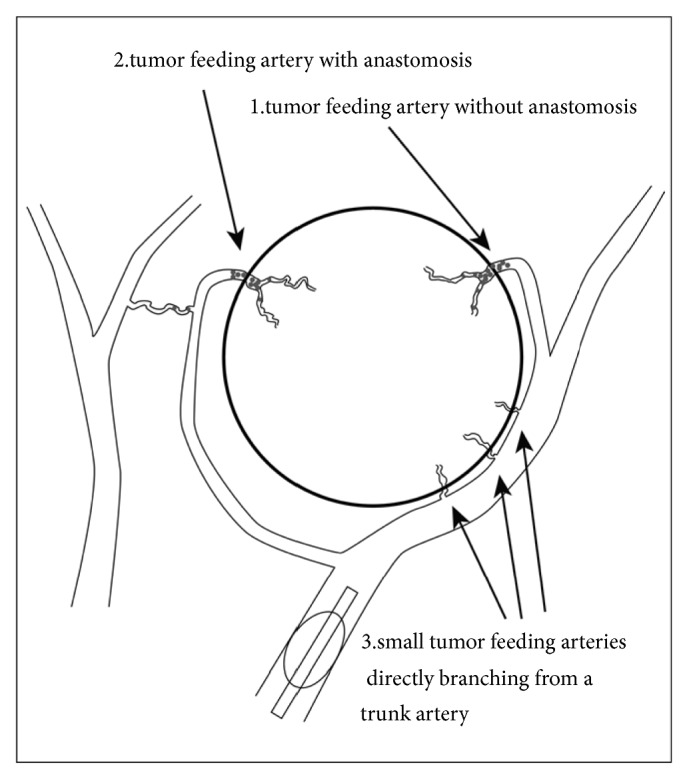
Hypothesis to explain the mechanism of RAIB-TACE. When proximal portion of trunk artery is occluded with a balloon, cisplatin solution and gelatin slurry flow into tumor feeding arteries without anastomosis at first (1). Then, they are pushed into tumor feeding arteries beyond the anastomosis (2). Near end-point of arterial flow cease beyond the microballoon catheter, cisplatin solution is concentrated into small tumor feeding arteries directly branching from a trunk artery (3).

Basing on arterial occlusion process during RAIB-TACE procedures (Figure 5) on DSA and fluoroscopy, we here propose a hypothesis to explain the mechanism of RAIB-TACE (Figure 6) as follows: (1) when proximal portion of hepatic artery was occluded, blood was also supplied to the nodule via multiple collateral arteries [14–17]. (2) The sizes of collateral arteries were various and anticancer drug solution and gelatin slurry flowed into arterial branches with no or low collateral flow at first. (3) Arteries with high collateral flow were embolized by subsequent gelatin infusion. (4) Near end-point of embolization, infused cisplatin would be concentrated into very small arteries which could not be embolized with gelatin slurry. Finally, all tumor feeding arteries beyond the micro-balloon catheter were treated while enclosing highly concentrated cisplatin in the nodule and preventing aggregation of gelatin fragments at proximal portion. To prove the presence of very small tumor feeding arteries invisible on angiography, microvascular casting and scanning electron microscopy of the surgical specimen are required.

As described previously [6], it was also difficult to control nodules adjacent to GS by Lip-TACE in our study. The difficulty in control of nodules adjacent to GS was well known, but publication of papers about this issue was limited [6]. Even when super-selective catheter placement in small tumor feeding arteries could be achieved, neither CR nor PR could be achieved in some cases by Lip-TACE (Figure 1). We speculated that smallness, tortuosity, and multiplicity of tumor feeding arteries directly branching from the trunk artery would be the causes of this Lip-TACE-refractoriness. On fluoroscopic observation, lipiodol droplets were far larger than the diameter of small tumor feeding arteries and lipiodol droplets tended to flow into peripheral liver parenchyma. The surface tension of lipiodol droplets worked to keep its shape round and prevented them to flow into small arteries directly branching from the trunk artery.

Efficient infusion of drug and/or embolization material into these tumor feeding arteries would be the key point for control of small nodules adjacent to GS. Placement of a microballoon catheter at the trunk artery would enable infusion of lipiodol emulsion into these smaller tumor feeding arteries (Figure 2), but we did not infuse much lipiodol in such cases to prevent atrophy of liver parenchyma. Compared with peripherally located small nodule that is not in close contact with GS, small nodule adjacent to GS was centrally located. Thus, more volume of peripheral liver parenchyma was influenced by lipiodol when TACE was performed via the trunk artery. On fluoroscopic observation, lipiodol droplets tended to flow into peripheral liver parenchyma. So, efficient infusion of lipiodol emulsion into small arteries directly branching from a large trunk artery might be difficult, we speculated. In RAIB-TACE, cisplatin was used as solution, and it could flow into small tumor feeding arteries like blood. Use of sparse gelatin slurry prevented aggregation of gelatin fragments, and small tumor feeding arteries could be efficiently embolized in RAIB-TACE technique.

Sparse gelatin slurry was not used for Lip-TACE because sparse gelatin slurry contained much volume of fluid and the lipiodol in the nodule would be washed out into the portal venous system when infused until arterial flow could be ceased.

Although the frequency of biloma by Lip-TACE was unknown when nodules associated with biliary dilatation was treated, it was a relative contra-indication as described by others [21]. Before development of RAIB-TACE, we had performed single shot transarterial infusion of anticancer drug without embolization, but neither PR nor CR could be scarcely achieved. We performed RAIB-TACE for 2 Lip-TACE refractory nodules associated with slight biliary dilatation at first, and CR could be achieved for both nodules without biloma (Figure 1). Then, we decided to include nodules associated with slight biliary dilatation for RAIB-TACE treatment. Nodule adjacent to GS has a potential of biliary dilatation by compression/invasion with the nodule, and development of treatment strategy was an important issue. Actually, 5 of 14 nodules were associated with slight biliary dilatation in this study, and RAIB-TACE caused biliary damage in 2 of 5 (40 %). However, the risk of RAIB-TACE for nodules with more dilated biliary tract was still unknown.

Biloma and stricture of right hepatic bile duct were seen in RAIB-TACE group, but no biliary complication in any 29 in Lip-TACE group. However, it could not be concluded that the risk and incidence of biliary damage was higher in RAIB-TACE because both nodules in RAIB-TACE were associated with slight biliary dilatation and Lip-TACE was not performed when the targeted nodule was associated with biliary dilatation. But we should remind the potential risk of biliary damage when nodules adjacent to GS with biliary dilatation were treated by RAIB-TACE.

Cisplatin was used for RAIB-TACE. We expected it as the most appropriate drug for RAIB-TACE because it was used most frequently for transarterial infusion treatment of HCC [22, 23]. Other drugs such as doxorubicin, epirubicin, and mitomycin C have not been examined for RAIB-TACE.

In this study, only small nodules 4 cm or less were analyzed. However, depending on this analysis, we ceased super-selective lipiodol TACE/B-TACE to treat large nodules since December 2014 and performed RAIB-TACE because large nodules were supplied by more tumor feeders compared with small ones and/or frequently located adjacent to GS.

There are several limitations in this study. (1) This study was a retrospective one. To evaluate the safety and efficacy of RAIB-TACE and to estimate whether overall survivals could be prolonged or not, randomized prospective comparative study was necessary. (2) Biliary damage occurred in 2 of 5 nodules associated with slight biliary dilatation. But we could not reveal why biliary damage occurred in 2 cases due to smallness of study population.

In conclusion, our new technique, “RAIB-TACE,” improved tumor response for patients with small HCC nodules adjacent to the Glisson sheath compared to Lip-TACE for similar HCC nodules. RAIB-TACE was also effective for HCC nodules unresponsive to Lip-TACE. RAIB-TACE may cause biloma or biliary stricture in patients with HCC nodules associated with localized biliary dilatation. A prospective, randomized clinical trial is necessary to evaluate the safety and effectiveness of RAIB-TACE for HCC nodules of less than 4 cm.

## Figures and Tables

**Table 1:** Details of patients and evaluation of side effects basing on common terminology criteria for adverse events (version 4).

	RAIB-TACE (n=12)	Lip-TACE (n=29)	p Value
Age (year)	76.0 ± 8.6 (62-86)	74.1 ± 10.0 (52-90)	0.56 1)
Male: Female	10:2	22:7	0.70 2)
Child-Pugh score (A:B)	10:2	28:1	0.20 2)
Hepatitis (C:others)	7:5	24:5	0.12 2)
Past history of RFA	7	17	1.0 2)
Past history of TACE	5	12	1.0 2)
Elevation of AFP or PIVKA II 4)	9	22	1.0 2)
Elevation of ALT (grade 1:2:3) 5)	10:2:0	26:2:1	0.66 3)
Elevation of Bilirubin (grade 1:2)	5:7	11:18	1.0 2)

1) Student's t-test.

2) Fisher's test.

3) Mann-Whitney *U* test.

4) AFP: alpha-fet protein (>20 ng/mL), PIVKA II: protein induced by Vitamin K absence or antagonists-II (>40 mAU/mL).

5) ALT: alanine aminotransferase.

There was no significant difference in any factors of patient's profile or side effects between the groups.

**Table 2:** Size and response of nodules.

	RAIB-TACE (n=14)	Lip-TACE (n=29)	p value
Diameter (mm)	23.5 ± 8.2 (12-39)	18.0 ± 7.8 (7-37)	0.064 1)
Tumor response (CR+PR:SD+PD)	13+1:0	17+1:4+7	0.008 2)

1) Student's t-test.

2) Fisher's test.

Two patients in RAIB-TACE group had 2 nodules adjacent to PSG.

Better tumor response was seen in RAIB-TACE group.

**Table 3:** Response of Lip-TACE refractory nodules.

CR:PR:SD:PD	7:0:2:0

CR was achieved in 7of 9 Lip-TACE refractory nodules by RAIB-TACE.

## Data Availability

Data availability could not be accepted by the ethics committee of the hospital. The clinical data used to support the findings of this study have not been made available because no allowance of review board of each hospital to disclose the data is obtained.
